# Supplementary material for: What does it take to learn the rules of RNA base pairing? A lot less than you may think
Source: bioRxiv. 2025 Aug 2:2025.07.31.668042. Preprint. [Version 1] doi: 10.1101/2025.07.31.668042 (PMC12324431; doi:10.1101/2025.07.31.668042)
Supplement: Supplement 1 [file media-1.gz › supplemental_material/experiments/g6_optimize_param_conus_rnabench_RNaseP.25.A_uniform/g6_params_i71.pdf]

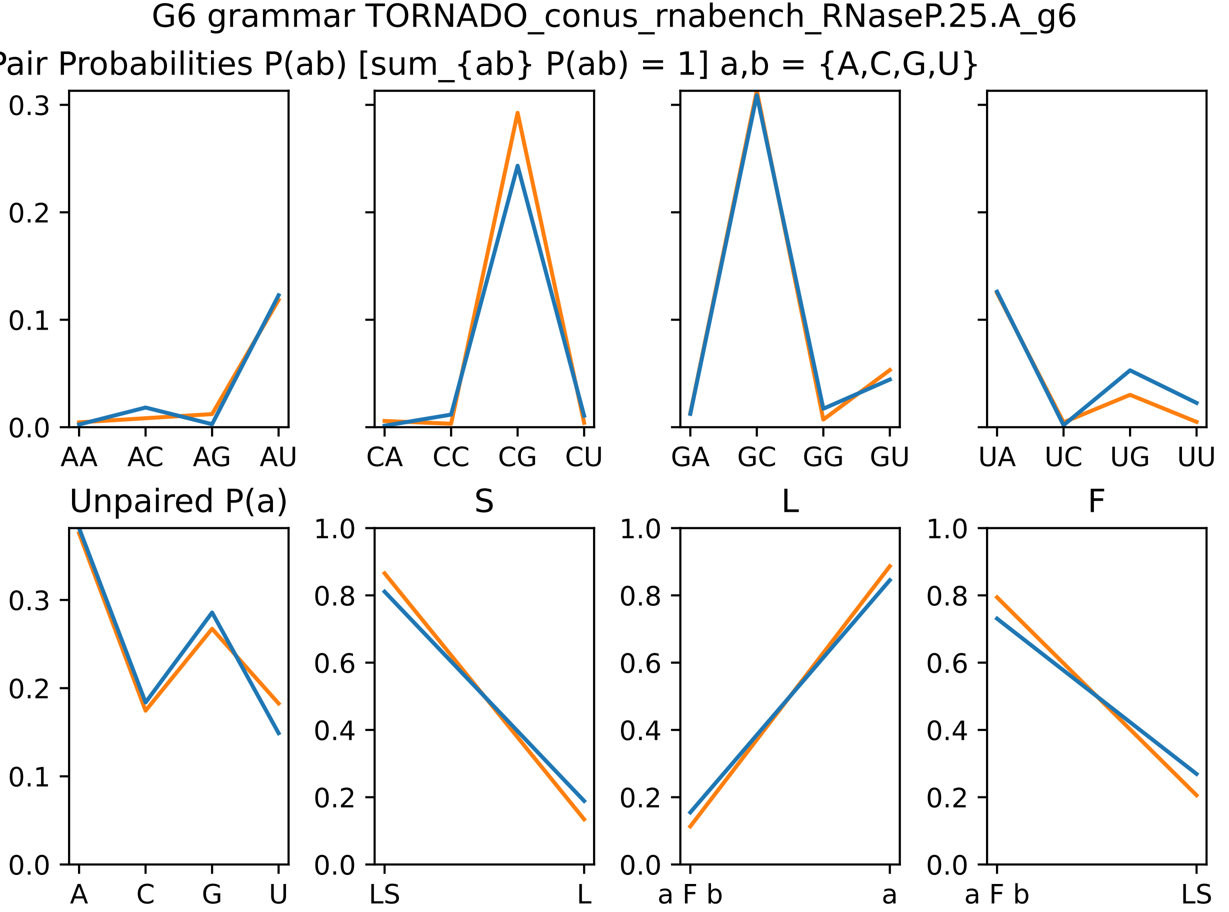
G6 grammar TORNADO_conus_rnabench_RNaseP.25.A_g6
Pair Probabilities P(ab) [sum_{ab} P(ab) = 1] a,b = {A,C,G,U}
0.3
0.2
0.1
0.0
AA AC AG AU
CA CC CG CU
GA GC GG GU
UA UC UG UU
Unpaired P(a)
A C G U
S
LS L
L
a F b a
F
a F b LS
1.0
0.8
0.6
0.4
0.2
0.0
